# The effects of a botanical anti-inflammatory nutritional supplement while participating in a resistance training program on indices of body composition and metabolic, cardiovascular, muscular, and hemodynamic function in obese females

**DOI:** 10.1186/1550-2783-12-S1-P40

**Published:** 2015-09-21

**Authors:** Sarah McKinley-Barnard, Josh Gann, Tom Andre, Erika Knue, Darryn S Willoughby

**Affiliations:** 1 Exercise and Biochemical Nutrition Lab, Department of HHPR, Baylor University, Waco, TX 76798, USA

## Background

Botanical supplements with flavonoids possess the ability to reduce inflammatory markers such as CRP, IL-6, and TNF-α. Also, they could potentially help reduce sugar-induced weight gain and facilitate weight loss. Diafin is a non-stimulant, botanical, weight loss product created from a blend of standardized Free-B-ring flavonoids and flavans from two plant extracts isolated from the *Scutellaria *genus of plants and the *Acacia *genus of plants. Flavonoids, specifically from the *Scutellaria *genus, have been used previously for anti-inflammatory and cardiovascular applications, and have been suggested to inhibit eicosanoid generating enzymes such as phospholipase A_2, _cyclooxygenases, and lipoxygenases, while concomitantly reducing prostanoids and leukotrienes. However, the exact mechanism in which flavonoids induce an anti-inflammatory effect is unclear.

## Purpose

The purpose of this study was to determine the effects of eight weeks of daily ingestion of a botanical, anti-inflammatory, nutritional supplement combined with resistance training and an energy-controlled diet on body composition, muscular performance, and serum lipids, obesity hormones, and inflammatory markers.

## Methods

Sedentary, obese women (n = 40) participated in a full-body resistance training program 3 days/week for 8 weeks while following an energy-restricted, low-glycemic diet and also ingested either 125 mg of a botanical, anti-inflammatory product (Diafin, Unigen Pharmaceuticals, Lacey, WA) or 125 mg of a cellulose placebo in a randomized, double blind, placebo-controlled fashion. Body composition, muscle performance, serum lipids, and inflammation and obesity markers were obtained at week 0 and after weeks 4 and 8. Data were analyzed by repeated measures ANOVA and are presented as means ± SD.

## Results

For body composition, there was a significant time main effect for body mass, BMI, and fat mass. Body mass (p < 0.001), BMI (p < 0.001), and fat mass (p = 0.034) all decreased significantly for both groups between weeks 0 and 8. For muscle performance, there was a significant time main effect for leg press and bench press strength as both strength variables increased in both groups between weeks 0 and 8 (p < 0.001). For serum lipids, there was a significant time main effect for TCHOL, LDL, and HDL. TCHOL (p = 0.004), LDL (p = 0.048), and HDL (p = 0.009) decreased between weeks 0 and 8. There was also a significant time main effect for leptin, which decreased significantly between week 0 and 8 (p = 0.019).

## Conclusion

It is concluded that a full-body resistance training program, in combination with an energy-restricted, low glycemic diet: 1) promotes weight loss and strength gains, 2) improves total and LDL cholesterol, and 3) decreases circulating leptin levels in previously-sedentary, obese women.

